# Sternal Pseudoaneurysm After Cardiac Surgery

**DOI:** 10.1016/j.jaccas.2025.103525

**Published:** 2025-06-04

**Authors:** Sarah Hickman, Ansab Fazili, Katherine Parkin, John Yap, Bhavna Pitrola, Mahrukh Qureshi, Amir Awwad

**Affiliations:** a St Bartholomew’s Hospital, Barts Health NHS Trust, Department of Radiology, London, United Kingdom; b Royal Papworth Hospital NHS Foundation Trust, Department of Radiology, Cambridge, United Kingdom; c Institute of Cardiovascular Science, University College London, London, United Kingdom

**Keywords:** doppler ultrasound, internal mammary artery, pseudoaneurysms

## Abstract

**Background:**

This report reviews a rare case of a presternal pseudoaneurysm after cardiac surgery.

**Case Summary:**

An 80-year-old male patient presented with a large lump over the manubrium at the upper end of his sternal wound. Two months previously, the patient electively received a tissue aortic valve replacement, and ascending aorta repair for an incidental 6-cm ascending aorta aneurysm and aortic regurgitation. Ultrasound examination revealed a pulsatile Doppler flow (swirling yin/yang) in a 2- × 6-cm lump, confirming the presence of a pseudoaneurysm. On arterial computed tomography imaging, the presternal pseudoaneurysm was found to be arising from a branch of the right internal mammary artery. The patient underwent urgent open repair, with the cause found to be rubbing of a residual sternal wire on the right internal mammary artery.

**Discussion:**

The case highlights the key imaging findings of sternal pseudoaneurysms after cardiac surgery and also provides a literature review of surgical and endovascular management.

## Graphical Abstract

**Figure:**
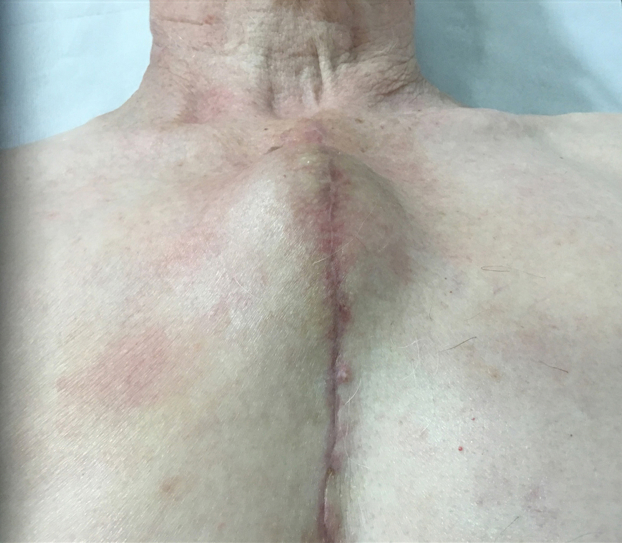

Pseudoaneurysms are defined as a discontinuity in the vascular wall, without a true vascular wall (false aneurysm), and may only have a hematoma cap preventing exsanguination. Each internal mammary artery (IMA) arises from the first part of the respective subclavian artery and is a paired artery running lateral to the sternum, terminating at the musculophrenic and superior epigastric arteries. Although pseudoaneurysms in aortic disease and after aortic surgery are not uncommon, presternal involvement of the IMA is rare. Previous case reports have identified these aneurysms arising due to trauma (penetrating or blunt), iatrogenic causes (eg, poststernotomy, coronary artery bypass, or pericardiocentesis), and infection (tuberculosis, actinomycosis, and *Staphylococcus aureus*). True aneurysms could form due to underlying connective tissue disorders (eg, Ehlers-Danlos, Loeys-Dietz, Marfan syndromes, and fibromuscular dysplasia) or idiopathic causes.^1^, ^2^, ^3^, ^4^

**Take-Home Messages**

• This case highlights a rare complication of a sternotomy with the formation of a presternal internal mammary pseudoaneurysm and its associated imaging findings.
• A literature review highlighted there is no consensus on open vs endovascular repair but highlighted the different approaches that can be taken for repair as well as why an open approach was chosen in this case.

A previous case report from 2009 explored a series of iatrogenic IMA pseudoaneurysms since 1973 to identify a total of 10 cases in the literature.^2^ With a mean age of 60 years old, 3 patients were surgically explored for bleeding/wound infection, at an average time interval of presentation of 4 weeks postoperation. Most cases presented as a subcutaneous mass that underwent open repair.^2^ Subsequent case reports have shown similar demographics and presentations.^5^, ^6^, ^7^, ^8^ Without appropriate treatment, these pseudoaneurysms can either rupture, leading to hemothorax, hemoptysis, hemorrhagic shock, or become infected with further fatal outcomes.^3^^,^^4^

## History of Presentation

An 80-year-old male patient underwent a routine repair of an ascending aortic aneurysm measuring 6 cm and an aortic valve regurgitation, found on routine echocardiography. On preoperative transesophageal echocardiogram, the patient was also found to have a fenestrated secudum atrial septal defect and small patent foramen ovale. The procedure was carried out via a median sternotomy (background of moderate pectus excavatum) to replace the ascending aorta with an interposition tube graft and tissue aortic valve replacement, and to repair both atrial septal defect and patent foramen ovale. The sternum was then closed using 6 straight double Mayo wires while the soft tissue was closed using layered absorbable Polyglactin (Vicryl) sutures. The patient was on clopidogrel; apixaban was added before discharge for postoperative atrial fibrillation. The patient had an unremarkable recovery except for the postoperative atrial fibrillation.

**Figure 1:**
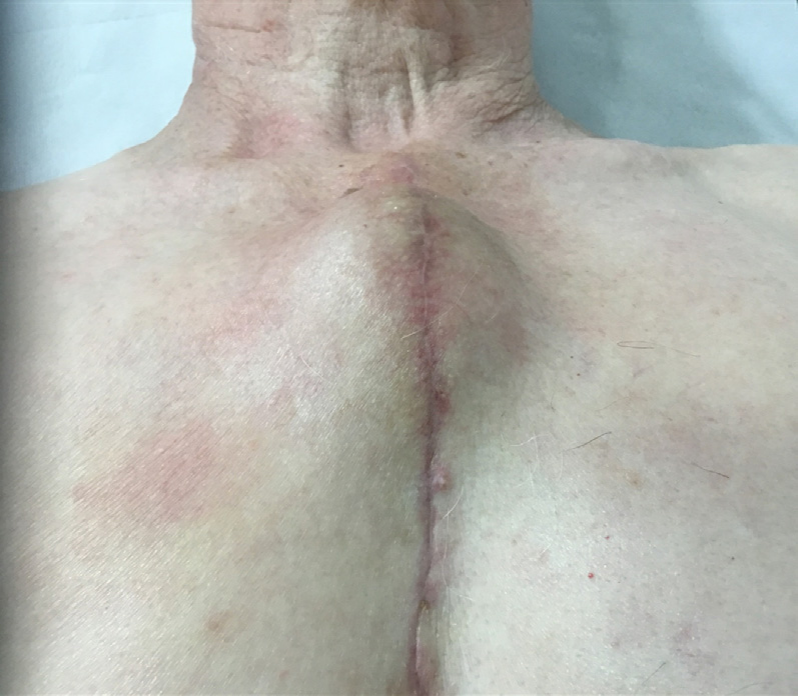
Clinical Photograph Clinical photograph of the large globular mass at upper portion of the midline sternotomy scar at presentation 2 months postoperation.

Two months later, the patient presented to the wound clinic with a painless, slowly growing lump overlying the manubrium at the upper part of the sternotomy wound (Figure 1). The lump was nonpulsatile, hard, and slightly tender. The patient was otherwise well.

## Past Medical History

There was a past medical history of hypertension, transient ischemic attack, and chronic kidney disease. There was no signs or symptoms of an underlying connective tissue disease.

## Differential Diagnosis

The differential diagnosis for a poststernotomy swelling may include abscess, localized hematoma, or benign seroma.

## Investigations

**Figure 2:**
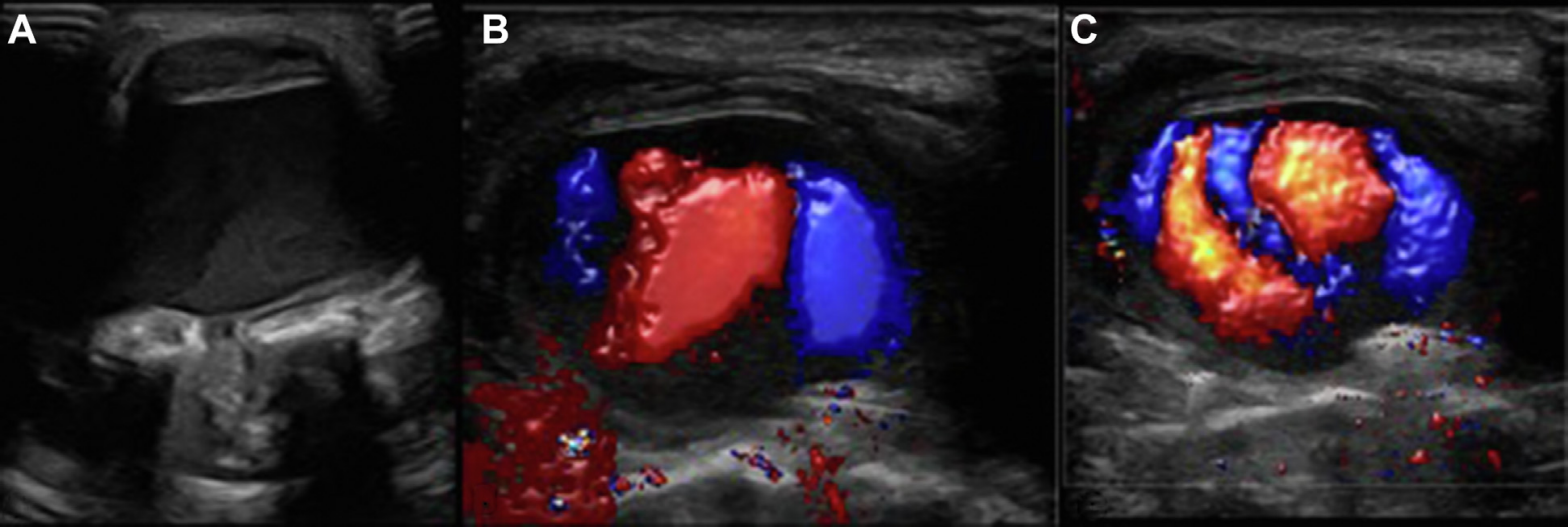
Ultrasound Images Ultrasound images on initial presentation of the upper sternal lump 2 months postoperatively. (A) B-mode imaging shows a wave at the inferior aspect of the sac indicating flow within the sac, (B and C) Color Doppler imaging demonstrates the yin/yang sign of swirling flow in a pseudoaneurysm.

**Figure 3:**
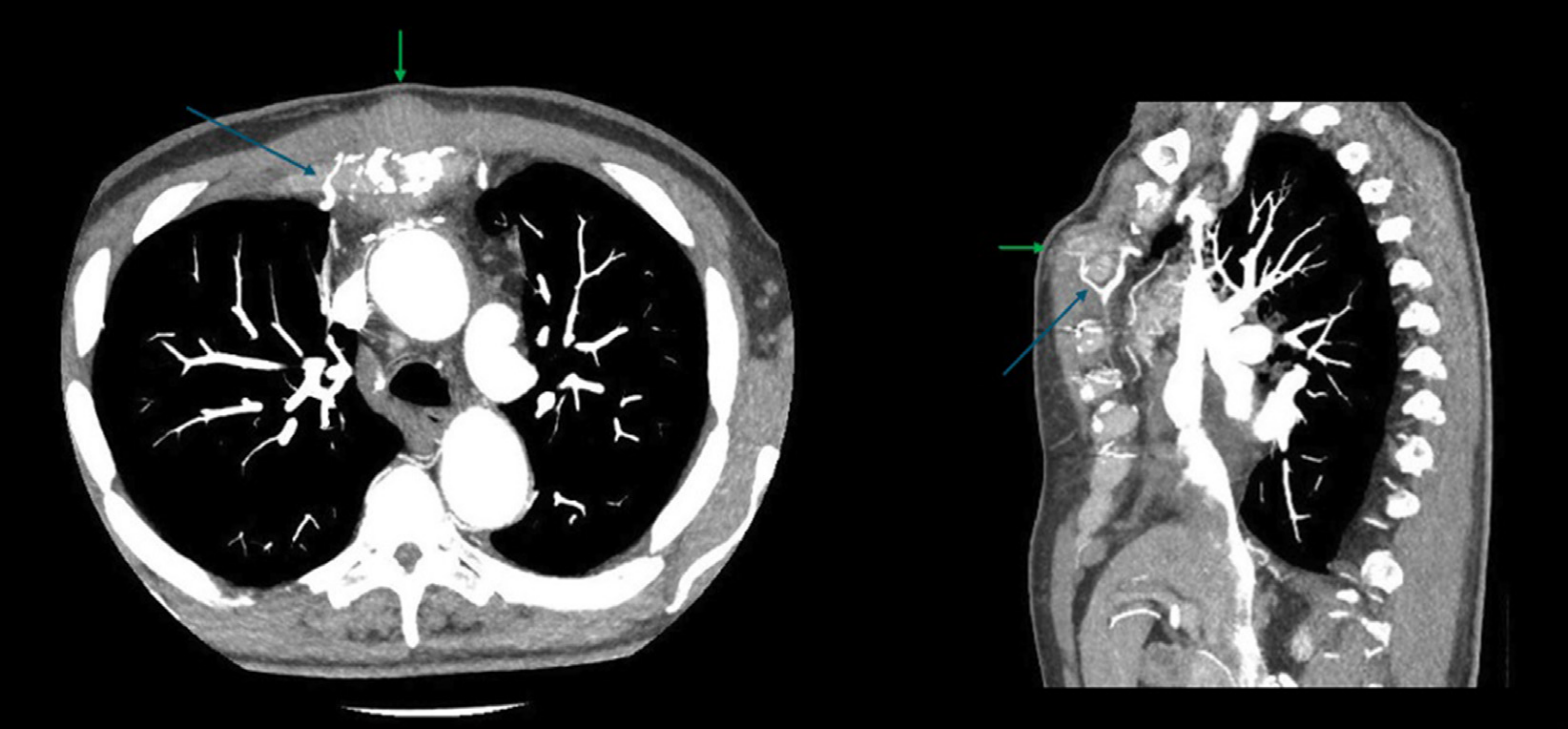
Enhanced CT Angiogram Enhanced CT angiogram in the arterial phase with maximum intensity projection (MIP) of axial (right) and sagittal (left) images, demonstrating a small feeding vessel from the right IMA (blue arrow), supplying the pseudoaneurysm sac in the midline anterior to the sternum (green arrow). CT = computed tomography; IMA = internal mammary artery.

A referral was made for a routine outpatient soft tissue ultrasound. Under ultrasound examination at the site of the upper sternum wound, the lump corresponded to a 2- × 6-cm well-defined hypoechoic area, representing a swelling with internal flow on B-mode imaging. Color Doppler showed dual flow within the sac in a typical yin/yang sign of dynamic swirling flow in keeping with a pseudoaneurysm (Figure 2, Videos 1 and 2).^9^ On computed tomography (CT) imaging, a horizontal branch of the right IMA was demonstrated to course anteriorly and supply the pseudoaneurysm sac (Figure 3).

## Management

Subsequently, the patient underwent an open repair of the sternal pseudoaneurysm. Intraoperatively, it was noted that the first sternal wire had injured the right IMA leading to the pseudoaneurysm formation. This sternal wire was removed, the pseudoaneurysm was resected, and all bleeding points were oversewn with multiple pledgeted ethibond sutures and muscular compression.

## Outcome and Follow-up

The patient swiftly recovered, and was discharged with no further complications. At the 6-week follow-up the patient continued to be well with no evidence of recurrence and no need for any further imaging.

## Discussion

Reviewing the literature since 1973, full access to 18 case reports on iatrogenic IMA pseudoaneurysms formation (Table 1) was granted. Previous case reports provide clear insight into the course and descriptive elements behind these pseudoaneurysms, from presentation to outcome (Table 1). The most frequent symptom reported by patients was the swelling itself, although a small number of cases presented initially with a hemathorax, a known consequence of pseudoaneurysm rupture. Notably, only roughly a third of reported cases were studied with ultrasound. A rare case of a “coin-shaped lesion” identified on a chest radiograph, proven to be a pseudoaneurysm, has also been conveyed in the literature. The case reports discussed open, endovascular interventional radiology repair (coil, glue embolization, or stent placement) as well as thrombin injection of IMA pseudoaneurysms (Table 1).^1^^,^^2^^,^^6^^,^^10^ Ultimately, all published cases reported uneventful recoveries with no documented ongoing complications.

**Table 1:** Summary of Previous Case Reports for Iatrogenic Internal Mammary Pseudoaneurysm Formation: Outlining the Initial Operation, Presentation, and Management

Number	First Author (Year)	Age, y/Sex	Previous Intervention/ Surgery	Presentation	Time to Presentation	Location	Imaging	Hemorrhage (Yes/No)	Treatment	Outcome
1	Martin (1973)	42/F	Median sternotomy for mitral valve replacement	Small, soft swelling on third left intercostal space lateral to sternum	29 d	LIMA	CXR	No	Surgical repair	NA
2	Den Otter (1978)	30/F	Augmenting mammoplasty	Coin-shaped lesion on CXR	Within 3 y	LIMA	CXR	No	Surgical repair	Uneventful recovery
3	Millner (1991): Patient 1	68/F	Median sternotomy for excision of left atrial myxoma	Expansile mass in right 5^th^ intercostal space	4 wk	RIMA	CT chest + contrast, USS, angiography	No	Surgical excision and RIMA ligation	NA
4	Millner (1991): Patient 2	69/M	Median sternotomy for aortic valve replacement	Hot pulsatile swelling along right sternal edge	4 wk	RIMA	CT	No	Surgical excision and RIMA ligation	Uneventful recovery
5	Agathos (1993)	51/M	Redo sternotomy for CABG	Angina	4 wk	LIMA graft	Lateral CXR, angiography	No	Suture ligation	Uneventful recovery
6	Frank (1998)	65/M	CABG, Robiscek Weave	Shock	7 wk	RIMA	CXR, CT chest + contrast	Yes	Emergency thoracotomy and suture ligation	Uneventful recovery
7	Callaway (2000)	69/M	Redo sternotomy for mitral and aortic valve replacements	Pulsatile mass over left border of sternum	2 mo	LIMA	USS doppler, angiography	No	Endovascular covered coronary artery stent	Uneventful recovery
8	Kamath (2005)	45/M	Emergency median sternotomy and prosthetic aortic valve replacement	Hemothorax	NA	RIMA	CXR, CT chest + contrast	Yes	Selective coil embolization	Satisfactory progress
9	Nasir (2009)	70/F	Median sternotomy for mitral valve replacement	Rapidly expanding chest wall mass	4 wk	RIMA	USS doppler, CT angiography	No	Selective coil embolization	Uneventful recovery
10	Cheung (2013)	85/M	Redo sternotomy, aortic valve replacement and repeat CABG	PEA arrest	21 d	LIMA	CXR, CT chest	Yes	Endovascular covered stent	Uneventful recovery
11	Mehra (2014)	78/F	Pericardiocentesis	Sudden-onset pain and swelling of left breast	7 d	LIMA	USS breast, NC CT chest, TTE, DSA	Yes	Microcoil + gel-foam embolization	Uneventful recovery
12	Falconieri (2015)	78/F	Redo sternotomy for mitral valve repair, tricuspid annuloplasty and left atrial appendage closure	Parasternal swelling	8 wk	LIMA	USS chest, CT angiogram	No	Selective embolization	Uneventful recovery
13	Datta (2016)	56/M	Coronary artery bypass and sternal wound infection with vacuum-assisted closure therapy	SOB and hypotension	10 d	RIMA	CT angiogram	No	Surgical excision	Uneventful recovery
14	Jefferson (2017)	71/F	Midline sternotomy for aortic valve replacement	Right-sided chest wall pain and swelling	8 wk	RIMA	CT angiography	No	USS-guided thrombin injection	Uneventful recovery
15	Al-Radhi (2018)	59/M	Aortic valve replacement	Right parasternal pulsatile mass	8 wk	RIMA	CT chest with contrast	No	USS-guided thrombin injection	Uneventful recovery
16	Koruda (2020)	49/F	Median sternotomy for redo aortic root replacement	Rapidly developing hemothorax	3 d	RIMA	CXR, CT, angiography	Yes	Selective coil embolization	Uneventful recovery
17	Inoue (2021)	71/F	Mitral valve replacement	Pulsatile lump in anterior thorax	NA	LIMA	CT chest + contrast	No	Selective coil embolization	Uneventful recovery
18	Hamdan (2022)	78	Mitral and aortic valve replacement and annuli repair for infective endocarditis	Rapidly growing parasternal mass	2 mo	RIMA	CXR, USS doppler	No	Surgical drainage of cavity and ligation of feeder artery	Uneventful recovery

References for the table can be found in the Supplemental Appendix.

CABG = coronary artery bypass graft; CT = computed tomography; CXR = chest x-ray; DSA = digital subtraction angiography; F = female; LIMA = left internal mammary artery; M = male; NA = not available; NC = non contrast; PEA = pulseless electrical activity; RIMA = right internal mammary artery; SOB = shortness of breath; TTE = transthoracic echocardiogram; USS = ultrasound.

There is no current consensus on the most ideal management of these pseudoaneurysms, although there may be now a trend away from surgical repair with advancements in endovascular/interventional solutions. With endovascular solutions becoming more commonplace, high-resolution (thin slices) enhanced CT imaging allows for precise anatomical evaluation (including access site, vessel caliber, and tortuosity as well as any anatomical variations) and appropriate operative planning. If a feasible endovascular repair is performed, this often occurs in 2 stages: first by embolization of the pseudoaneurysm sac, followed by a later second stage surgical procedure to evacuate the hematoma, as necessary. Overall, the less-invasive endovascular repair could minimize bleeding, shorten hospital stays, and reduce anesthetic complications.

Reviewing the literature, the most common method of repair remained surgical with 44.4% (8 of 18) of cases using this method across all time periods from 1973-2022, as well as our case in 2024. However, the literature does show a trend away from surgical repair in the early 2000s with the use of coil embolization being the second most common method reported 33.3% (6 of 18), with 1 case combining the use of gel foam and coil embolization. A less common method of endovascular repair included the use of covered stents used in 11.1% (2 of 18) of cases. Endovascular covered stents can be used if there is sufficient space to land a stent without covering a major branch. Both coiling and stenting techniques are well established for visceral pseudoaneurysms rather than iatrogenic superficial types.

Ultrasound -guided thrombin injection is a type of minimally invasive treatment that has largely replaced ultrasound -guided compression and is commonly used for the treatment of femoral artery pseudoaneurysms, due to a shorter procedure time and reduced pain for the patient. This is the method of choice for small persistent or larger yet asymptomatic pseudoaneurysms. For a successful procedure, long- and narrow-necked aneurysms are preferred. Patients are monitored for 4-6 h postprocedure and a follow-up ultrasound in 24 h is recommended to monitor for resolution. For pseudoaneurysms that are enlarging or become acutely symptomatic, interventional or surgical treatments are recommended. The use of thrombin injection for the treatment of iatrogenic IMA pseudoaneurysms was reported in 11.1% (2 of 18) of cases in our literature review and appears to be becoming more common in recent years with cases reported in 2017 and 2018.

The differential diagnosis, as detailed already in this paper, for a poststernotomy swelling may include abscess, localized hematoma, or benign seroma. Alternatively, any swelling with a small focal oozing skin break or hole that may correspond to a superficial enhancing tract on CT imaging would raise the sinister suspicion of an aorto-cutaneous fistula. In the context of a sternal swelling poststernotomy, care must be taken to perform the correct imaging before needle intervention, which could risk fatal complications.

In this case, the pseudoaneurysm formation was due to a small vascular injury as a result of repeated arterial wall irritation by the first sternal wire. Open repair here was thought to be the most appropriate remedy because it explored the depth of the problem and allowed for the sternal wire to be removed at the same time. Discussion regarding the need for endovascular repair included if there was an open access issue or difficulty (eg, pseudoaneurysm pointing internally toward the heart), however, this was not required in this case.

## Conclusions

This case detailed a postsurgical sternotomy pseudoaneurysm formation from repetitive trauma of the first sternal wire to an anterior branch of the right IMA, which is an uncommon event from a branch of the IMA, and was treated with open repair. Similar nontender rapidly growing and persistent sternal swellings would warrant careful clinical observation and accurate assessment with Doppler and CT (arterial) imaging. The report also exhibits our management outcome with background literature review of different open surgical and endovascular interventional radiological therapy of such cases.

## Funding Support and Author Disclosures

The authors have reported that they have no relationships relevant to the contents of this paper to disclose.

## Appendix

Video 1 Cine file showing dynamic swirling flow in the pseudoaneurysm in B mode and with doppler color flow.

Video 2 Cine file showing dynamic swirling flow in the pseudoaneurysm with color doppler flow.

Table References
