# Supplementary material for: Sternal Pseudoaneurysm After Cardiac Surgery
Source: JACC Case Rep. 2025 Jun 4;30(13):103525. doi: 10.1016/j.jaccas.2025.103525 (PMC12235142; doi:10.1016/j.jaccas.2025.103525)
Supplement: Table References [file mmc3.docx]

References for Table 1

1. Martin A, Ross BA, Braimbridge M V. Peristernal wiring in closure of median sternotomy. False aneurysm of the internal mammary artery. J Thorac Cardiovasc Surg 1973;66(1):145–6.

2. Den Otter G, Stam J. Aneurysm of internal mammary artery. Thorax 1978;33(4):526–7. <https://doi.org/10.1136/thx.33.4.526>.

3+4. Millner RWJ, Guvendik L, Blauth C, Treasure T, Pepper JR. False aneurysm of the right internal mammary artery. Ann Thorac Surg 1991;51(5):831–2. <https://doi.org/10.1016/0003-4975(91)90144-F>.

5. Agathos EA, Hussein A, Trehan H, Trenholme SE, Floten HS. Traumatic pseudoaneurysm of left internal mammary artery graft. Ann Thorac Surg 1993;56(4):966–8. <https://doi.org/10.1016/0003-4975(93)90366-P>.

6. Frank MW, Alexander JCJ, Pineless GR, Votapka T V, Curran RD. False aneurysm of the right internal mammary artery. Late rupture after sternotomy. Texas Hear Inst J 1998;25(1):86–7.

7. Callaway MP, Wilde P, Angelini G. Treatment of a false aneurysm of an intercostal artery using a covered intracoronary stent graft and a radial artery puncture. Br J Radiol 2000;73(876):1317–9. https://doi.org/10.1259/bjr.73.876.11205677.

8. Kamath S, Unsworth-White J, Wells IP. Pseudoaneurysm of the internal mammary artery as an unusual cause of post-sternotomy hemorrhage: The role of multislice computed tomography in the diagnosis and treatment planning. Cardiovasc Intervent Radiol 2005;28(2):246–8. https://doi.org/10.1007/s00270-004-4195-y.

9. Nasir A, Viola N, Livesey SA. Iatrogenic Pseudoaneurysm of Internal Mammary Artery: Case Report and Literature Review. J Card Surg 2009;24(3):343–5. <https://doi.org/10.1111/j.1540-8191.2009.00838.x>.

10. Cheung PKM, Philipp RK, Freed DH. Endovascular stenting for treatment of a left internal mammary artery pseudoaneurysm following redo-sternotomy: A case report. Catheter Cardiovasc Interv 2013;82(5):778–81. <https://doi.org/10.1002/ccd.24850>.

11. Mehra S, Buch A, Truong CN, Moshiri M, Shriki JE, Bhargava P. Post-traumatic internal mammary artery pseudoaneurysm: A rare complication of pericardiocentesis. Radiol Case Reports 2014;9(1):931. <https://doi.org/10.2484/rcr.v9i1.931>.

12. Falconieri F, Raevsky E, Davies S, Moat N. Pseudoaneurysm of a branch of left internal mammary artery: A late and potentially fatal complication after redo-sternotomy. Interact Cardiovasc Thorac Surg 2015;20(6):866–7. <https://doi.org/10.1093/icvts/ivv059>.

13. Datta S, Manoly I, Karangelis D, Hasan R. Pseudoaneurysm of the Right Internal Mammary Artery Post Vacuum-Assisted Closure Therapy: A Rare Complication and Literature Review. Ann Vasc Surg 2016;31:207.e1-207.e3. https://doi.org/10.1016/j.avsg.2015.08.010.

14. Jefferson J, Nuffer Z, Butani D. Thrombin Injection to Treat an Iatrogenic Internal Mammary Artery Pseudoaneurysm. J Med Ultrasound 2017;25(3):177–9. <https://doi.org/10.1016/j.jmu.2017.08.001>.

15. Al-Radhi Y, Shrivastava V. A novel approach to treating a post-sternotomy internal mammary artery pseudoaneurysm. Interact Cardiovasc Thorac Surg 2018;26(1):161–2. <https://doi.org/10.1093/ICVTS/IVX254>.

16. Kuroda Y, Uchida T, Hamasaki A, Sadahiro M. Internal thoracic artery pseudoaneurysm after redo aortic root replacement. Cardiovasc J Afr 2020;31(5):281–2. <https://doi.org/10.5830/CVJA-2020-014>.

17. Inoue K, Kainuma S, Kashiwagi E, Toda K, Sawa Y. Post-Sternotomy Internal Mammary Artery Pseudoaneurysm. JACC Cardiovasc Interv 2021;14(4):e37–8. <https://doi.org/10.1016/j.jcin.2020.11.037>.

18. Hamdan R, Jazayeri S, Terriat B, Melin-Boucetta I. Iatrogenic False Aneurysm of the Internal Thoracic Artery after Sternotomy: The Role of Doppler Ultrasound. Vasc Spec Int 2022;38:1–2. <https://doi.org/10.5758/vsi.220043>.
